# The holistic development of talented sportspersons through dual-career

**DOI:** 10.3389/fspor.2022.929981

**Published:** 2022-07-28

**Authors:** Ricardo T. Quinaud, Laura Capranica, Mojca Doupona, Flavia Guidotti

**Affiliations:** ^1^Physical Education Department, University of the Extreme South of Santa Catarina, Criciúma, Brazil; ^2^Department of Movement, Human and Health Sciences, University of Rome “Foro Italico”, Rome, Italy; ^3^Department of Sport Sociology, Faculty of Sport, University of Ljubljana, Ljubljana, Slovenia

**Keywords:** student-athlete, education, athletes' career, sport, career support

## The holistic development of talented athletes

In sport, talent development is a multidimensional, multiplicative, and dynamic interaction of performance, psycho-social, and educational processes (Simonton, 2001; Burgess and Naughton, 2010). In general, the development of talents spans a period of 15–20 years and encompasses different stages in the sport (e.g., initiation, talent development, talent retention, mastery, and perfection/elite performance), education (e.g., elementary school, high school, and university/vocational), and working levels (Stambulova and Wylleman, 2015, 2019; Wylleman, 2019; Moreno et al., 2021). Therefore, in pursuing an athletic career several decisions must be taken, which have a direct impact on the lives of the sportspersons and their academic/sport/family and peers supporting entourage (Ryba et al., 2015; Capranica and Guidotti, 2016; Condello et al., 2019; Gjaka et al., 2021; Leisterer et al., 2021; Stambulova et al., 2021; Tessitore et al., 2021; Varga et al., 2021). Also, the literature on sports talent identification emphasises the importance of significant actors for facilitating the holistic development of talented athletes, mainly through supportive initiatives/actions in pursuing wellbeing and in coping with stressful situations across life domains, such as training and competitive loads, injuries, lack of social life, and transitions to higher competition and academic levels (Morgan and Giacobbi, 2006; Johnston, 2018; Williams and MacNamara, 2022). In revisiting its position stance on athlete transitions and wellbeing, the International Society of Sport Psychology (ISSP) specifically highlighted career assistance as a crucial aspect (Stambulova et al., 2021). During talent development and career transitions, it is important to collect information on the athletes' lifestyles, relationships, and supportive entourage to plan and apply effective interventions.

During their developmental stages, youth athletes encounter increasing demands and challenges, also in relation to concurrent interactions between sport and education requirements (Salmela-Aro et al., 2008; MacNamara and Collins, 2010; Aquilina, 2013; Monteiro et al., 2017). In particular, to build a mastery elite performance level in their sport, student-athletes competing in different sports disciplines spend several hours in training, competition, and sport-related side activities (i.e., warm-up, cool down, recovery interventions), to be combined with academic commitments such as class attendance and individual study (Ericsson, 2006; Aquilina, 2013; Guidotti et al., 2015; Condello et al., 2019). The time spent in training, travelling to competitions, and competing poses athletes in a disadvantaged position compared to their non-athletic counterparts (European Commission, 2012; Xanthopoulos et al., 2020). In fact, athletes reported a lack of time to study, limited relations with teachers/professors, classmates, and peers, missed classes and exams, physical and mental fatigue, and identity conflicts (Gaston-Gayles and Baker, 2015; Stambulova et al., 2015; Gomez et al., 2018; Condello et al., 2019; Steele et al., 2020). Despite athletes having the main responsibility of their dual career paths (e.g., micro dimension) several individuals, institutions, or specific contexts have different and integrated responsibilities in accompanying and fostering talented athletes during their developmental years, mainly providing a critical balance of challenges and/or emotional and logistical support at the meso (e.g., parents, peers, teachers/employers, coaches, sport managers), macro (e.g., sports clubs/federations, educational institutions, and labour market), and policy (e.g., national and international governing bodies) dimensions of dual career (Larsen et al., 2012, 2013; Capranica and Guidotti, 2016).

## Challenge for scholars on the dual career of athletes

In the last decade, several aspects have been studied to uncover the dual-career phenomenon (Guidotti et al., 2015; Stambulova and Wylleman, 2019; López-Flores et al., 2021). However, the actual interpretation of findings is limited by country-, sport-, and academic-specific socio-economic-cultural contexts, which determine tremendous differences in dual-career regulations, programmes, and services. In particular, the researchers tend to use the term student-athlete, which strictly refers to a sports context rooted in an educational system (e.g., United States) and could present some problems when applied to athletes as students competing in sports organisations not related to academic institutions (e.g., Europe). Another critical aspect pertains to relevant differences in the requirements and eligibility criteria for dual-career programmes and services adopted within and across countries, which determine unequal quantity and quality of dual career support (European Commission, 2016). Finally, the definition of a dual career as “a career with major foci on sport and studies” (Stambulova and Wylleman, 2015, p. 1) could allow different interpretations when the sport or the academic careers are not balanced or linear over time, and when strict academic and sport eligibility criteria are adopted, supporting a short-term approach to outcomes rather than long term holistic development of athletes (Martindale et al., 2005; Staurowsky and Sack, 2005; Capranica and Millard-Stafford, 2011). This lack of clarity has a direct influence on how scholars analyse and interpret findings, and suggests cautions when ways to support dual-career paths of talented athletes are envisaged.

Considering that the holistic development of youth athletes is a complex process involving different individuals, organisations, and socio-cultural-political systems, qualitative and quantitative multilevel mixed methods research designs are recommended to advance our understanding of the interactions occurring at its micro, meso, macro, and global levels (Headley and Plano Clark, 2019). In fact, the use of multilevel analysis lies in the fact that it considers the nature of data structure and the different sources of variation (Gelman and Hill, 2006). Furthermore, in light of the extensive globalisation of sport and the internationalisation of educational paths, the scientific community is urged to cooperate in establishing evidence for the implementation of dual-career guidelines for an effective sport- and academic-specific support of youth talented athletes. Besides the academic community, also the athletes and their supportive entourage, the managers, the policy-makers, and the stakeholders are required to contribute with innovative and cooperative approaches for the holistic development of the youth athletes. In this framework, the successful experience of the European Commission to allocate funds for cross-national cooperation through the ERASMUS+Sport Collaborative Partnerships focused on dual-career and youth development provides a valuable example for the establishment of a platform for fostering evidence- and eminence-base knowledge uncovering effective bidirectional relationships between policies and practises (Guidotti et al., 2015; Stambulova and Wylleman, 2019; Capranica et al., 2021; European Commission, 2021; López-Flores et al., 2021).

## The responsibilities and challenges in the dual-career micro dimension

Talented athletes committed to achieving high performances in their sports might need effective proactive strategic planning to facilitate their transition to the elite level as well as to the labour market at the end of their sports career. Indeed, several individual aspects concur with a holistic developmental programme, including a deep understanding of the athletes' potential profiles in relation to the dynamic association of their endogenous (e.g., physical and mental traits, and personal values) and exogenous (e.g., cultural and physical environments) resources, as well as a sound understanding of potential barriers (Gagné, 2013; Simonton, 2017; Weissensteiner, 2017). Undoubtedly, intrapersonal characteristics could help define a strong student-athlete identity, motivation, willpower, and time management, which could improve the probability of successful dual-career paths (Li and Sum, 2017). Whilst athletes competing in championships managed by a sports federation or associations and being enrolled in a full-time high school or university degree could consider themselves student-athletes, different eligibility criteria are adopted to allow them to access institutionalised dual career services and provisions (Capranica and Guidotti, 2016: European Commission, 2016; Sanchez-Pato et al., 2017). Furthermore, individual self-identity and motivation to combine academic and sports careers differ based on the athletic level, sports career perspectives, self-awareness, and personal values of the athletes, as well as the dual-career support from cultural backgrounds and contexts (Gaston-Gayles, 2005; Harrison et al., 2014; de Subijana et al., 2015; Lupo et al., 2015; Quinaud et al., 2021; Lee et al., 2022). Therefore, the initial identification of future sports and academic performances would be based on a thorough understanding of the most relevant determinants supporting dual career paths and preventing risks of sports or academic burnout and dropouts. Finally, interventions might occur to implement the development of a dual career supporting entourage for the athletes.

## The responsibilities and challenges at the dual-career meso dimension

The meso dimension of dual-career comprises actors having strong, direct, and personal relationships with the athlete in the family (e.g., parents, siblings, relatives, friends, and peers), the sport (e.g., coaches, managers, staff, dual-career tutor), and the academic (e.g., classmates, teachers, tutors, deans) environments. In particular, elite athletes competing at the 2017 summer Universiade (e.g., the world's largest and most prestigious multi-sport events organised for university athletes by the International University Sports Federation–FISU) declared that parents, coaches, and university staff are their best dual-career supporters (Condello et al., 2019). In fact, parents play a key role in the climate created for sports and education, whereas coaches and teachers increase their role as the athletes grow older and relocate to academies, or experience language barriers and cultural adjustment when migrating abroad (Baghurst et al., 2018; Fuchs et al., 2021; Palumbo et al., 2021). Parents, coaches, and teachers could have a concurrent and additive role in the athlete's outcomes and wellbeing when aligning objectives for the promotion of a holistic developmental process for the athletes. However, cooperation between coaches and parents of young athletes is not promoted (Capranica and Millard-Stafford, 2011; Knight et al., 2018; Mossman et al., 2021; Lemelin et al., 2022).

Recent research focusing on the parent's role in sustaining athletes' dual-career highlighted difficulties in establishing meaningful relationships with sports and academic staff for the construction of a coherent dual-career support environment (Gjaka et al., 2021; Tessitore et al., 2021; Varga et al., 2021). In considering that parents may lack the required knowledge to work individually and in teams with other key dual-career actors, a European framework informed the development of an online education programme for parents within the Erasmus+ Sport project EMPATIA to empower them in promoting a positive dual-career environment for their talented children (Capranica et al., 2018, 2022; Varga et al., 2021). To avoid the mutual interference between educational and athletic environments, academic and sport, staff might consider rethinking their role through appropriate formal (e.g., degree programs) non-formal (e.g., refreshment courses), and informal (e.g., reading, conversations with experts) training opportunities. Additionally, staff may consider a cultural-specific approach to integrating professional, interpersonal, and intrapersonal knowledge. The integration of such knowledge will contribute to establishing a climate of listening, questioning, and negotiation between dual-career actors to develop and/or support a team of facilitators of an effective development environment for talented athletes (European Commission, 2020; Neelis et al., 2020; Nikander et al., 2022).

## The responsibilities and challenges in the dual-career macro dimension

The holistic development of individual and team sports athletes have a multi-centric organisational model encompassing sports bodies (e.g., clubs/national sports federations, athletes' organisations), educational institutions (e.g., schools/universities), and service provider of well-structured and coherent programmes at school and at sports levels that recognise athletes to be seen as whole persons (Capranica and Guidotti, 2016). Around the globe, the organisation of sports and education varies considerably in structure, typology, and administration, ranging from models embedding sport in the educational system to sports practised in clubs having no or limited relationship with the educational system (Camiré, 2014; European Commission, 2016).

At the sports level, national Olympic Committees and sports federations/associations could adopt a top-down approach by promoting dual-career programmes through educational courses for coaches and managers, and by fostering the inclusion of a dual career tutor at club levels to facilitate collaboration with educational institutions. Furthermore, athletes' organisations could adopt a bottom-up approach by requesting that clubs and sports bodies adopt measures in support of dual careers of youth talents for their holistic, integrated, and sustainable development. At the academic level, in the United States a well-structured dual career is in place, with the educational provision (e.g., scholarships, academic tutors, career counselling, etc.,) used as a strategic tool to recruit the most talented athletes for upholding high school and university sports teams considered symbols of academic institutions (www.nfhs.org; www.ncaa.org). In Europe, sports are mainly organised at the club level and there is a need for specific dual-career national guidelines and regulations to avoid a fragmented and incoherent culture to support youth athletes towards their achievements in the sport and academic domains (Aquilina and Henry, 2010; De Bosscher et al., 2011, 2015; European Commission, 2012, 2016; Henriksen et al., 2014, 2020; Thomsen and Nørgaard, 2018; Kuettel et al., 2020; Morris et al., 2021; Nikander et al., 2022). Even when the athletes have been considered symbols of their schools, their academic performances have been an issue of concern. Despite there being no consensus regarding the negative influence of sport on graduation rates and academic success, the negative impact of stereotypes on the academic underperformance of athletes urge the creation and implementation of identity-safe environments (Jonker et al., 2009; Levine et al., 2014; Storm and Eske, 2021; Storm et al., 2021; Hsu et al., 2022).

To behave authentically, the sports and academic environments may need to pro-actively translate dual-career values into their own actual practises and to ameliorate strategically their processes and practises. In particular, we recommend to academic institutions: i) guarantee the recognition of the “student-athlete” status based on pre-defined criteria characterising elite sports-persons (e.g., enrolment in the National Team, sport professionalism, number of certified hours of training per week, certified competition level); and ii) provide the necessary services (e.g., flexibility in class attendance and examination schedule, tutoring/consulting, on-line learning opportunities) to meet athletes' needs to combine their sport and academic efforts. Similarly, sports organisations and coaching staffs may need to recognise the educational demands athletes' have to match with their training and competition schedule and to provide them concrete support in their dual career path (e.g., sports facilities and services, training schedule adaptation when possible, and proximity between sport and academic venues). Therefore, the alignment of sports and academic institutional efforts and strategies at both internal (e.g., education of the staff members, change in the processes of dual career management) and external (e.g., collaborative practises for the establishment of coordinated dual-career programmes through the involvement of all the relevant dual-career stakeholders) levels is crucial to develop and support athletes' dual career (Capranica and Guidotti, 2016). Despite a positive relationship between sports bodies and educational institutions being strongly envisaged to determine effective dual career paths, it is crucial to consider that no single programme or best practises implemented in specific settings could be generalised across national contexts, sports disciplines, and educational environments. Thus, tailored strategic inter-institutional agreements on dual-career support have to be designed, monitored, and evaluated over time (Emrich et al., 2009; Jonker et al., 2009; Henriksen et al., 2014; Thompson et al., 2022).

## The responsibilities and challenges in the dual-career policy dimension

In addition to personal and organisational efforts in advancing dual-career values, understanding, and beliefs, also sports federations, governments, and societal expectations have a role in sustaining the advancement of the dual-career culture at both national and international levels through specific policies and financial resources (Capranica and Guidotti, 2016; Kuettel et al., 2018). At the international level, the European Parliament (2003, 2015, 2021), the European Commission (2012, 2021), Council of the European Union (2021), the Council of Europe (2021), the International School Sport Federation (ISF, 2022), the International University Sports Federation (FISU, 2021), and the International Olympic Committee (IOC, 2022), have a top-down influence and provide the framework for cross-national and cross-sectoral cooperation between decision-makers. Their recommendations could foster the identification and the promotion of the best practises in dual-careers at local, national, and international levels, as well as overcome the resilience of the educational and sports institutions that might not envisage the need for changes. In fact, to counteract the lack of education in favour of sports commercialisation during the athletes' developmental years, the recent Resolution of the Council of the European Union on a European Model of Sport (2021) and the Recommendations of the Council of Europe on the Revised European Sports Charter (2021) urge policymakers and sports stakeholders to stress the development of the youth and the rights of the child and to invest in education through sports (Council of Europe, 2021; Council of the European Union, 2021). Furthermore, the allocation of funds to cross-sectorial and cross-country partnerships and to studies focused on dual-career and youth development practises could accelerate the development of a culture supporting the holistic development of youth athletes (Guidotti et al., 2015; Stambulova and Wylleman, 2019; Capranica et al., 2021; European Commission, 2021; López-Flores et al., 2021).

Additionally, International and National guidelines on dual careers could further enhance major tenets and praxis for social support for the holistic development of talented athletes in different sports and educational settings (European Olympic Committee, 2011; European Commission, 2012). Moreover, the European Athlete as Student (EAS) network provides a platform for an effective dialogue between educational bodies (i.e., universities, high schools, sports schools), sports organisations (i.e., clubs, sports federations, National Olympic Committees), and cooperates with European institutions (e.g., European Parliament, European Commission, and Council of Europe) and several partners in the development of innovative international cross-national projects and research on a dual career in the diverse contexts, which represent a laboratory for reconciling youth sport and education also beyond Europe (Aquilina and Henry, 2010; Capranica et al., 2015, 2021; Capranica and Guidotti, 2016; Condello et al., 2019; Stambulova and Wylleman, 2019; López-Flores et al., 2021).

## Conclusion

Despite the primacy and independence of sports and education policies and legislation, in the past decade, there is a growing concern to sustain the athlete's right of combining sport and academic careers and to identify the relevant factors that impact the nature of support provision and the level of disruption leading to a sport or academic drop-out (Henry, 2013). At present, the evidence indicates that no single individual, variable, or model effectively ensures the sound development of talented athletes (Guidotti et al., 2015; Stambulova and Wylleman, 2019; López-Flores et al., 2021). Consequently, extensive cooperation between public authorities, sports bodies, academic institutions, and other stakeholders is strongly recommended to promote opportunities for the implementation of dual-career guidelines (European Commission, 2012; Mittag et al., 2021). Furthermore, sports scholars are urged to increase the clarity of definitions of terms and to apply innovative, multidisciplinary, and cross-national research approaches for envisaging proper strategies that enhance the holistic development of talented athletes.

Scientific evidence could help overcome some resistance due to stereotypes privileging education over sports to prepare for a future life, or privileging sports over education to obtain outstanding athletic outcomes. Several issues not fully operationalized in the literature might need further investigations to verify: The impacts of financial resources on the athletes' development; the actual sports and academic outcomes of athletes receiving qualified dual-career measures; the implementation of dual-career programmes resulting from educational programmes for sports and academic staff; and the monitoring and evaluation measures to implement the efficacy of dual-career development environments. In fact, in considering the increased socio-cultural expectations of supporting talented athletes, the combination of education and sport is not sufficiently implemented to facilitate favourable dual-career environments (European Commission, 2016).

## Author contributions

All authors contributed intellectually to the conceptualisation and the actual writing-up of this opinion paper.

## Conflict of interest

The authors declare that the research was conducted in the absence of any commercial or financial relationships that could be construed as a potential conflict of interest.

## Publisher's note

All claims expressed in this article are solely those of the authors and do not necessarily represent those of their affiliated organisations, or those of the publisher, the editors and the reviewers. Any product that may be evaluated in this article, or claim that may be made by its manufacturer, is not guaranteed or endorsed by the publisher.
